# Everyday life consequences of substance use in adult patients with a substance use disorder (SUD) and co-occurring attention deficit/hyperactivity disorder (ADHD) or autism spectrum disorder (ASD): a patient’s perspective

**DOI:** 10.1186/s12888-014-0264-1

**Published:** 2014-09-19

**Authors:** Linda M Kronenberg, Karin Slager-Visscher, Peter JJ Goossens, Wim van den Brink, Theo van Achterberg

**Affiliations:** Department of residency training MANP mental health, Dimence, P.O. Box 50037400 GC, Deventer, The Netherlands; Dual Diagnosis Department, Dimence, Deventer, The Netherlands; Assertive Community Treatment, Dimence, Deventer, The Netherlands; Saxion University of Applied Sciences, Expertise in Centre of Health, Social Work & Technology, Deventer, The Netherlands; SCBS, Dimence, Deventer, The Netherlands; Scientific Institute for Quality of Healthcare, Radboud University Nijmegen Medical Centre, Nijmegen, The Netherlands; Amsterdam Institute for Addiction Research, Academic Medical Center University of Amsterdam, Amsterdam, The Netherlands; Centre for Health Services and Nursing Research, KU Leuven, Leuven, Belgium; Department of Public health and Caring Sciences, Uppsala University, Uppsala, Sweden

**Keywords:** Substance use disorders, Attention deficit hyperactivity disorder, Autism spectrum disorder, Everyday life consequences, Adults

## Abstract

**Background:**

Although the prevalence of substance use disorder (SUD) with co-occurring attention deficit/hyperactivity disorder (ADHD) or autism spectrum disorder (ASD) is relatively high in adult patients, there is hardly any knowledge about these dual diagnoses. A recent study reported met- and unmet needs for several life domains regarding these patient groups. To improve treatment, it is necessary to identify the everyday life consequences of SUD and co-occurring ADHD or ASD in adult patients.

**Methods:**

Qualitative study using in-depth interviews. 11 SUD + ADHD and 12 SUD + ASD patients participated in the study. The interview transcripts were coded and analysed according to the seven steps for descriptive phenomenology by Colaizzi.

**Results:**

Both patients with ADHD and patients with ASD can get caught in a jumble of thoughts and emotions which can often lead to agitation and impulsivity in the case of ADHD or passivity and melancholia in the case of ASD with co-occurring SUD in both cases. Initially substance use ameliorates the symptoms and related problems, but both patient groups can later experience even greater problems: difficulties with the structuring of daily life due to a lack of planning (SUD + ADHD) or due to a lack of initiative (SUD + ASD). Both groups indicate that structure helps them function better. They also recognize that substance use disorganizes their lives and that an absence of structure contributes to substance use in what becomes a vicious circle which needs to be broken for effective treatment and care.

**Conclusions:**

This study provides insight into the daily life consequences of SUD with a co-occurring ADHD or ASD. Substance use is reported to solve some ADHD- or ASD-related problems in the short run but have negative consequences in the long run (i.e., contribute to already impaired cognitive functioning). Insight is provided into what clinicians can do to break this vicious circle and thus help ADHD patients to refrain from action and ASD patients to take action.

## Background

Mental disorders can frequently co-occur with a substance use disorder (SUD) or some other disorder. Such co-occurrence is often referred to as a *dual diagnosis*, which can itself refer to either life-time co-occurrence or current co-occurrence. For clinical purposes and needs assessment, current co-occurrence is more important than life-time co-occurrence.

The co-occurrence of SUD and other mental disorders is very common: about 50% of individuals with severe and persistent mental disorders are affected by substance use [1-3]. Some studies have specifically examined the co-occurrence of SUD with schizophrenia, bipolar disorder, psychosis, depressive disorder or anxiety disorder [1,4,5]. Similarly, the co-occurrence of SUD with attention deficit/ hyperactivity disorder (ADHD) has been examined quite extensively. And a recent meta-analysis of the prevalence of attention-deficit hyperactivity disorder in substance use disorder patients showed 23% of all patients with SUD to meet the criteria for adult ADHD [6]. Studies of the co-occurrence of SUD and autism spectrum disorders (ASD) are scarce [7]. In recent research, however, the lifetime prevalence of SUD in connection with ASD has been reported to range from 11% to 29% [8-10]. The sample sizes have been limited (*n* = 122, *n* = 54 and *n* = 70 respectively) in these studies, however.

A very recent study showed about 1% of the general population to have adult ADHD with a co-occurring SUD while 0.1-0.2% of the general population has an ASD with a co-occurring SUD [11].

Studies have further shown the co-occurrence of SUD with other psychiatric disorders to be associated with not only ineffective treatment and care but also an unfavourable treatment course and outcome [12-14]. In a recent study the objective care needs of SUD patients with co-occurring ADHD or ASD were determined [11]. Met- and unmet needs were reported for several life domains and it was suggested that further research should focus on the (subjective) psychological consequences of substance use in SUD patients with ADHD (SUD + ADHD) or ASD (SUD + ASD). This calls for in-depth, qualitative study of substance use and related daily problems.

In light of the above, we undertook a qualitative, interview study to answer the following research question: What are the everyday life consequences of substance use in adult patients with a substance use disorder (SUD) and co-occurring attention deficit/hyperactivity disorder (ADHD) or autism spectrum disorder (ASD) from a patient’s perspective?

## Methods

### Study design and procedure

A qualitative, interview study was conducted among a population of treatment-seeking patients with a dual diagnosis of SUD and ADHD or SUD and ASD.

Open, in-depth semi-structured interviews were used in which patients in both groups were questioned about the consequences of their illness for everyday life using the patient’s perspective as the guiding principle. The study was approved by a certified medical ethics committee (Commissie Mensgebonden Onderzoek Regio Arnhem-Nijmegen) and by the institutional review board of Dimence (Commissie Wetenschappelijk Onderzoek). All participants signed informed consent for participation in the study.

The interviews were conducted first with a group of SUD + ADHD patients in the period of December 2011 to May 2012 and then with a group of SUD + ASD patients in the period of June 2012 to October 2012. All of the interviews were conducted by one of two researchers who thus conducted half of the interviews for each patient group. The interviews were then transcribed verbatim.

### Target patient population and selection criteria

Patients with SUD and a co-occurring ADHD or ASD were recruited using the following inclusion and exclusion criteria. Inclusion: outpatient treatment for SUD; age 18–65; IQ >80; current DSM-IV diagnosis of SUD and current DSM-IV diagnosis of ADHD or ASD; mastery of the Dutch language. Exclusion: diagnoses of SUD and both ADHD and ASD. All patients were recruited from an outpatient, dual diagnosis, treatment facility in the Netherlands.

Out of 122 patients, 72 told that they were willing to participate in the further study. Of these 72 patients, nine were diagnosed with *both* ADHD and ASD in addition to SUD and therefore excluded from further inclusion, leaving a total of 63 eligible patients.

A total of 37 SUD + ADHD patients were then approached for this study: seven patients could no longer be reached and 13 refused to participate despite their initial willingness to do so. An interview was thus planned for 17 patients, but 5 did not show up then. One patient also had to be excluded due to hospitalization at the time of the planned interview. This left 11 SUD + ADHD patients to participate in the study.

A total of 26 SUD + ASD patients were approached for this study as well: 5 could no longer be reached and 7 refused to participate despite initial willingness to do so. An interview was planned for 14 patients, but 2 of them did not show up. In the end, 12 SUD + ASD patients were interviewed for a total of 23 patients participating in the present study.

### Interview topics

The in-depth interviews were conducted with the aid of a topic list which draws upon the results of a previous-study [11]. *Impairments*, *unmet needs,* and *clearly met needs* were probed for a number of life domains (see Table 1).

**Table 1:** **Interview topic list (i.e., domains of life for which impairments and needs were probed)**

**Life domains indicated from previous study**	**Topics for present study**
Psychiatric health	
Psychological distress	Mental health
Mental health	
Drugs	
Alcohol	Addiction
Unemployment	
Day time activity	Daily activities
Looking after the home	
Family/social relations	
Company	Social functioning
Money	
Finances	Finance
Physical health*	Physical health
Sexuality**	Sexuality

*SUD + ADHD only.

**SUD + ASD only.

The topic list was used as guidance during the interview. By posing open questions, the patients were invited to describe in detail their ideas, attitudes, experiences, and behaviour. Sample questions were: *What are your problems like, and how do they relate to your alcohol or drug use? What does this combination of SUD and ADHD/ASD mean for you?* and *How does the combination of SUD and ADHD/ASD relate to your everyday life?*

### Coding and analysis of the interview transcripts

Given the qualitative nature of our study, the interview transcripts were coded and analyzed in a cyclic process. However, keeping the groups separated helped to focus on the in-depth knowledge within each group. Throughout this process, the routines followed and the coding procedures applied were discussed between the researchers and, in this manner, consistency of assessment was assured. The coding and analysis of the data was performed using the MAXQDA2010 software. And, in doing this, the seven steps for descriptive phenomenology outlined by Colaizzi [15] were followed.

In order to gain an overall impression of the data, the interview transcripts were first read as a whole (step 1). Then both interviewers independently selected and coded significant statements (steps 2 and 3). Related codes were then identified and grouped together under the themes they represented (step 4). Thereafter, a subset of 6 of the 23 interview transcripts was coded by both researchers and the findings compared.

After the completion of the first 7 interviews for each of the patient groups, an interim analysis was performed (step 5). The results of this analysis were then used to guide the subsequent interviews. The emerging themes and insights were thoroughly discussed in preparation for the subsequent interviews.

In the subsequent interviews, the themes identified in step 5 were used to determine whether new codes would emerge which could not be grouped within the existing themes or data saturation was reached.

In the next step of the analyses, the interviews from the two groups were examined as a whole but also compared to identify general and group-specific themes (step 6). The outcomes of the analyses were then summarized and sent to the participants for feedback and validation of the results (written member check, step 7). Unfortunately, none of the participants responded to our request for a written member check.

## Results

For both patient groups (SUD + ADHD N = 11; SUD + ASD N = 12), the need for care according to the EuropASI [11] was very similar to that of the total group of eligible patients (N = 63) for 5 of the 8 EuropASI domains. Only small differences were detected for the other 3 domains. More SUD + ADHD patients in the current sample were living alone and in both diagnostic groups in the current sample more patients were employed compared to the total eligible group. However, the differences were small and we thus conclude that the research groups were comparable to the total group of eligible patients.

The interviews with the patients lasted 60 minutes on average. The two patient groups were similar with regard to most socio-demographic characteristics; they only differed on gender composition and primary substance use. The SUD + ASD group included only males and 10 out of the 12 patients reported alcohol as their primary substance of use. The SUD + ADHD group included three women and eight men; primary substance use varied (see Table 2).

**Table 2:** **Demographic and clinical characteristics of two dual-diagnosis patient groups (n = 23)**

	**SUD + ADHD**	**SUD + ASD**
**Total** ***n***	**11**	**12**
Male *n* (%)	8 (73%)	12 (100%)
Age (mean)	43	37
***Living***		
Alone *n* (%)	6 (55%)	5 (42%)
With other(s ) *n* (%)	5 (45%)	7 (58%)
***Employment***		
Employed *n* (%)	6 (55%)	6 (50%)
***Substance of abuse*** *(more than 3 years*)		
Alcohol *n* (%)	2 (18%)	3 (25%)
Cannabis *n* (%)	-	1 (8%)
Alcohol + cocaine *n* (%)	3 (27%)	-
Alcohol + cannabis *n* (%)	1 (9%)	5 (42%)
Alcohol + medication *n* (%)	-	1 (8%)
Alcohol + cocaine + amphetamine + ecstasy *n* (%)	2 (18%)	-
Alcohol + medication + cocaine + amphetamine + cannabis *n* (%)	1 (9%)	-
Alcohol + heroine + cocaine + amphetamine + ecstasy *n* (%)	1 (9%)	-
Alcohol + cocaine + amphetamine + cannabis + XTC *n* (%)	-	1 (8%)
Extreme variety of substances (>6) *n* (%)	2 (18%)	1 (8%)

Comparison of the initial steps in the coding and analysis of the interview transcripts showed that the researchers had selected the same statements as significant, assigned largely similar content codes to the selected statements, and also grouped the coded statements into similar themes.

Following the seven-step analytic procedure, the everyday life consequences of a dual diagnosis of SUD and ADHD or SUD and ASD were found to revolve around three main themes: (1) *jumbled thoughts and emotions*, (2) *ambiguity of substance use, and* (3) *structure.*

In the following, we further describe the everyday consequences and supply illustrative quotations (with the patient group and respondent number indicated in parentheses).

### Jumble of thoughts and emotions

Both groups stated that they experience a jumble of emotions, including anger, distress and anxiety. Participants also said that they did not recognize emotions very well, and had difficulties understanding them. Furthermore, they were not able to handle their emotions and found it hard to sort them out and to put them into perspective. They often failed to make the connection between event and feeling, and vice versa.

*Distressed, sad, happy…. those things actually blend in my case, so to name how I’m feeling, that’s hard to do. (…) And that’s what sometimes happens to me, that I’m really not feeling good and things just get worse because I can’t figure out the reason why.* (SUD + ASD 2)

The jumble of emotions experienced by both groups of patients led to a vicious circle of negative thinking and feeling which led to vicious circle of symptoms and substance use. Both groups mentioned getting caught in this vicious circle and not being able to get out of it by, for instance, stopping the flow of negative thoughts and emotions.

*That’s because of us* [people with ADHD] *thinking that fast and much, and often we* [people with ADHD] *fill in many things by ourselves, and pick things up, a lot. That’s why things come on stronger to us* [people with ADHD] *and stick longer.* (SUD + ADHD 11)

The patients in the SUD + ADHD group frequently stated that they could not handle the flood of thoughts and emotions which they experienced. This could lead to agitation, which could trigger further impulsiveness. What is meant here is that patients cannot put the break on and therefore race past their own and others limits with serious negative consequences, such as using more addictive substances and/or experiencing more interpersonal conflicts.

In contrast, patients in the SUD + ASD group frequently expressed that the overload of stimuli caused by the jumble of thoughts and emotions made them passive and melancholic. This obviously had a negative impact on their quality of life. Feeling lonely and searching for some perspective in life are frequently mentioned by patients with SUD + ASD. In a relationship or similar contact, passive and melancholic feelings were also often reported to stand in the way of sexual activity.

*I always go on, and on, and on (…) you just get tired and more tired. The consequence of this is becoming less focused, and that makes you more and more impulsive.* (SUD + ADHD 11)

*Especially when I’m tired and worn out, I become very hyper (…) then you go bouncing. Becoming more inefficient. (…) what I’ll often do then, when I’m totally worn out, is use drugs … really lots of them....* (SUD + ADHD 7)

*I’ve got the idea that I can get lost in my head, in thought, which makes me lose time sometimes and that is what’s keeping me from getting to action.* (SUD + ASD 3)

*By now I know my mind is processing data very slowly, and if I want to live a pleasant and meaningful life then I’ll have to stop working as hard as I did before and I shouldn’t want to anymore. But that’s a hard nut to crack. (…) and to me that’s still intense and it really makes me worry about if I’ll get a girlfriend ever? Will I ever get to live with someone? (…) And sometimes that really makes me sad (…). It makes me feel lonely.* (SUD + ASD 2)

*I was longing for a relationship for a long time, because that’s the standard. However, it being the standard is not a very good reason to have a relation, I guess. (…) I’ve been in bed with a girl sometimes, and then I’m lying there and I just don’t know what to do, how to act, what’s normal.* (SUD + ASD 4)

### Ambiguity of substance use

Substance use was reported to serve a clear purpose for both of the patient groups. It suppressed the jumble of thoughts and emotions, the agitation, and the melancholia. For the SUD + ADHD group, substance use served to decrease the level of hyperactivity but was also often boundless: one bottle of beer led to the drinking of an entire case. For the SUD + ASD group, substance use was reported to help suppress the jumble of thoughts and emotions, to help them relax and get through the day, and to help them escape feelings of pure boredom.

*Giving myself a bit of peace, so it doesn’t get out of control. That my brain stops thinking, particularly. Stops going on, and on, and on.* (SUD + ADHD 8)

*Then we had children, and the stress got higher. (…) Well, I started drinking, just to inhibit the stimuli, to subdue them. (….) that helped me get through the evening. It was just to survive, not for the booze.* (SUD + ASD 3)

At the same time, the patients from both groups reported substance use to worsen their symptoms and the problems stemming from their ADHD or ASD.

*Especially when I had a relapse or used again, then I noticed becoming disordered for a long period. (…) and that’s when you let impulses rule you.* (SUD + ADHD 11)

*It has the effect that I don’t function properly, and that I hardly eat (…) Everything gets worse by using.* (SUD + ASD 10*)*

Stopping with substance use was reported to produce clear benefits by most of the SUD + ADHD patients. They experienced better physical and mental health as a result of quitting. They nevertheless indicated that quitting was a struggle because everything which their substance use had suppressed came to the surface … and then more intensively. They further observed that balanced use or controlled substance use in moderation was something which they could not manage and therefore not an option.

*I can’t restrain that, I just do it. I’ll blow, quaff or snort, there’s no restraint whatsoever. I can’t go and drink one beer, one beer becomes a case.* (SUD + ADHD 6)

One of the SUD + ASD patients reported that it had been relatively simple to stop and that he was not using anymore, but the remainder of the SUD + ASD group reported still using. These patients also reported searching for a way to achieve a kind of balanced use — mostly with professional guidance.

*… I have decided that I won’t be quitting drinking fully, because I noticed a great part of my social life will be gone and to me that’s not worth it, and I don’t know how it could be done without the booze.* (SUD + ASD 2)

### Structure

Both groups indicated that structure made them function better in many areas of life: daily activities, social relationships, finances, physical health, and sexuality. Having daily activities, a job, and family further helped them structure their lives and gave it meaning. Structure contributed to break out of passivity and control impulsiveness.

*But, if you get me sitting at home, then you’ve got another alcoholic, that doesn’t work out. To me, that’s my structure, my work and so forth.* (SUD + ADHD 5)

*A company for career counselling helps me to reintegrate in work, and I told them I don’t want to work in a big warehouse or with a boss or colleagues that will rouse me, because that will go wrong. And I don’t want to go back to my old lifestyle, that I’ll just have to smoke a joint in the evening.* (SUD + ASD 6)

*I noticed that when I neglect the housekeeping I also get more noise, more thoughts in my head (…) so I am very occupied with getting more discipline in housekeeping.* (SUD + ASD 2)

Both groups further noted — as indicated in the preceding and following quotations — that substance use disorganized their lives. In a vicious circle, *lack of structure* contributed to substance use and substance use contributed to a further lack of structure.

*When I don’t have a reason to get up in the morning (…) then I tend to stay in bed and take it easy. I’ll smoke a joint in the morning and will go back to sleep again. So, I noticed, I need structure in my life.* (SUD + ADHD 3)

*I find it hard to fill my time — I don’t have many hobbies — although structure is very important. It happened that I got so bored that it made me think: let’s drink tonight, so tomorrow I’ll sleep it off and then another half day is filled.* (SUD + ASD 5)

Patients in the SUD + ADHD group reported frequent problems with the actual structuring of activities. They mentioned knowing what they wanted to do, having the necessary skills for social relationships or to make plans, and believing that they could handle matters effectively. But they found it hard to prioritize, keep focus on the task at hand, and stay tuned into to not only their own needs but also the needs of others. The result was major difficulties with maintaining structure, sustaining relationships, and managing finances.

*I plan my time very inaccurately, and that takes a lot of energy, takes a lot of effort. (…) Yes, that’s the result. I get distracted by impulses, and I get distracted easily, and that is what makes you spend time on the wrong things.* (SUD + ADHD 11)

*I don’t know what to do if someone shares his emotions, that makes me become very nervous. So I’ll walk away. I had an affair with a woman for four years, and when things went wrong I walked away. I don’t know how to handle that. That’s not my cup of tea.* (SUD + ADHD 5)

*A lot of debts, once 3000 euros (…) because of my impulsivity, of course. Not because of using weed, no, I just bought all kinds of things.* (SUD + ADHD 10)

The SUD + ASD patients also experienced problems with structure but, in contrast to the SUD + ADHD group: they hardly knew how to spend their spare time, how to initiate a social relationship, and how to handle other matters; such as their finances or household.

*Things get into a routine, that’s what I don’t recognize. (…) It may happen that I don’t get myself to work or do other things for half a day (…) things seem to get stuck in my head, like a needle in the groove of a record, and I can’t get out of it.* (SUD + ASD 11)

*It doubles the handicap that you’ve got, makes it really hard to take stock of situations. And the drugs make you think even more … like … well … like passive. You get even more passive.* (SUD + ASD 6)

The thought of starting a social relationship or becoming socially active is reported to make most of the patients in the SUD + ASD group feel anxious and insecure. They reported not being able to easily join in with others and use substances to help them do this. Substance use suppressed feelings of insecurity and overstimulation/oversensitivity, made it easier for them to tune into people/situations, and helped them focus and react during interactions.

*But, when I have got to sit in sober, that really gives me the jitters.* (SUD + ASD 5)

*… it* [using alcohol] *makes it easier to put things in words, put it in words more quickly, react faster.* (SUD + ASD 3)

A number of the patients in the SUD + ASD group also reported lacking sufficient insight in their finance, because they did not take initiative and felt they were not able to keep track of their finances.

*I handed over financial and administrative matters to an administrator, voluntarily. In the past, those were daily activities that I didn’t get to. I noticed it, of course, that pile of bills lying there. But no, I was too scared to start on them.* (SUD + ASD 4)

Overall, the interviews showed the patients getting caught in a vicious circle of symptoms and substance use in every life domain.

## Discussion

**Figure 1:**
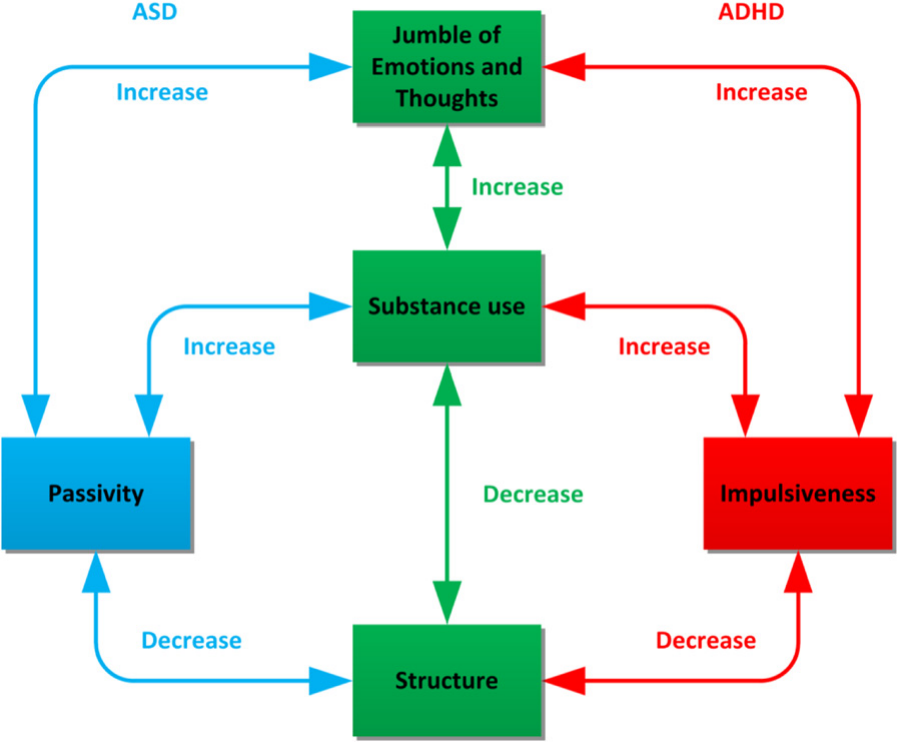
**Vicious circle entailed by dual diagnosis of SUD with ADHD or ASD.**

With regard to the everyday life consequences of SUD with co-occurring ADHD or ASD, it can be concluded that the *underlying mechanisms* appear to differ for the groups (i.e., impulsivity vs. passivity) but that the *everyday life consequences* of having a dual disorder are similar: both groups get caught in a vicious circle of symptoms and substance use. The cycle is more or less the same for the two groups: a jumble of thoughts and emotions; increased symptoms (i.e., impulsiveness or passivity); decreased structure; increased substance use; and occurrence of even more jumbled thoughts and emotions (see Figure 1). For both groups of patients, the jumble of thoughts and emotions can lead *directly* to agitation and substance use, which initially ameliorates the symptoms but exacerbates them later: for the SUD + ADHD group, substance use typically leads to increased impulsiveness; for the SUD + ASD group, substance use typically leads to increased passivity. In the long run, SUD increases the ADHD or ASD symptoms and interferes with self-management.

Both groups function better when there is sufficient structure. However, substance use destroys structure, and this lack of structure can lead to a vicious circle of increased substance use. The SUD + ADHD group of patients mainly lacks inhibition of impulsiveness and therefore has considerable difficulties focusing as well as maintaining structure. This created a situation of agitation and thereby increased substance use and heightened impulsivity, with a further loss of structure and increased substance abuse as an outcome. In contrast, the SUD + ASD group has problems with initiation, the management of daily responsibilities, and getting into and holding on to structure. The outcome is passivity and melancholia, which can lead to substance use as a form of coping (see Figure 1).

In their review, Kushner & Mueser [19] described four models explaining the high prevalence of comorbid substance use disorders and other psychiatric disorders; the common factor model, the secondary substance abuse model, the secondary psychopathology model, and the bidirectional model. The current study contains various examples of processes consistent with each of these models: impulsivity as a common vulnerability factor, substance abuse to reduce dysphoria or to facilitate social engagement, substance abuse leading to impulsive behavior or passivity, and - most of all - the presence of (bidirectional) vicious circles of increasing substance abuse and increasing psychopathology.

### Dealing with impaired executive functioning

A possible key to treatment of SUD with a co-occurring ADHD or ASD and thus breaking the vicious circle of symptoms and substance use is attention to their cognitive or so-called executive functioning (EF). The problems that are solved by EF are essential for adequate social functioning. EF is essential for adequate functioning in two main domains: inhibition and meta-cognition. Inhibition refers to the ability of the individual to inhibit motor, verbal, cognitive, and emotional activities. Meta-cognition refers to the individual’s nonverbal working memory, verbal working memory, planning abilities, problem-solving abilities, and emotional self-regulation [16].

For alcohol-dependent patients, research shows that almost all executive functions are impaired [17-21]. For the patients in our study and thus patients with a dual diagnosis of ADHD or ASD and a co-occurring SUD, an accumulation of EF impairments may thus be the case.

Barkley [16] recently found ADHD patients to have both impaired inhibition and impaired meta-cognitive functioning (i.e., both non-verbal and verbal working memory limitations). Not being able to concentrate or remember things for a longer period of time, patients with ADHD may move from task to task without re-engaging or finishing a task and thus leave a series of uncompleted tasks behind them. This is consistent with what we found in the present study for SUD + ADHD patients who reported being impulsive, problems focusing, trouble keeping focused, and difficulties maintaining structure in several areas of their lives. SUD may thus worsen the problems of patients with ADHD. This means an accumulation of EF impairments.

ASD is also associated with impairments of both inhibition and meta-cognition [22,23]. Patients with ASD have been shown to have problems with intention formation (i.e., performance planning), intention initiation (i.e., switching, time monitoring) and execution (i.e., task completion, switching, planning adherence, efficiency). Flexibility remains particularly impaired across ages in ASD, whereas working memory, initiation, and organization become increasingly problematic over time. SUD may thus worsen the problems for patients. This means an accumulation of EF impairments. This pattern of findings is consistent with what we detected in the present study for the SUD + ASD patients who reported being passive, difficulties with structure, and problems organizing various areas of their lives.

### Clinical implications

The results of this qualitative, interview study show how SUD creates a vicious circle of symptoms and substance use in patients with a dual diagnosis of SUD and ADHD or ASD. We also gained insight with this information into what clinicians can do to break the vicious circle of symptoms and substance use: SUD + ADHD patients should be helped to refrain from action and SUD + ASD patients should be helped to take action. Patients should be helped to create and maintain structure in their lives, and their self-management skills need to be strengthened to do this. In addition, SUD + ASD patients may initially be offered controlled substance use in the absence of appropriate behavioural alternatives for the realization of a treatment goal. A patient with SUD + ASD, for example, may be helped to engage in social activities with reduced drinking (i.e., controlled substance use) and thus to master the behavioural repertoire needed to participate in social activities [24].

Clinicians should nevertheless realize that reduced substance use or total abstinence will not always result in better planning, greater structure, or increased initiative. The cognitive impairments arising from SUD add to the often chronic, cognitive impairments associated with ADHD and ASD, which means that reductions in substance use may *help* but are not very likely to fully restore the cognitive functioning of individual with a dual diagnosis of SUD and ADHD or ASD. The goal of the treatment and care for these patients should thus be maximization of their long-term welfare — however the patient defines this — by helping them to break the vicious circle of symptoms and substance use. Support, reinforcement of available skills, and viable alternatives for cognitive impairments should always thus be considered.

### Strengths and limitations of the present study

The conduct of the interviews in the present study by two independent researchers increases credibility and reliability [25]. Analysis of the data was performed by the two researchers independently and final coding was based on consensus between the two researchers. Data saturation was reached when coding the statements selected from the transcripts for discussion topic, main themes, and relevant points, which indicates the validity of the study [26].

The group of responders and non-responders were comparable with regard to the intensity of care, which suggests that our patient population was representative. A limitation of the study was that the researchers did not use cross-over blind coding, which would have added to the reliability. Other possible limitations were that the SUD + ASD group included only males and that co-occurring mental health conditions aside from ADHD and SUD or ASD and SUD were not assessed.

## Conclusions

With regard to the everyday life consequences of SUD with co-occurring ADHD or ASD, it can be concluded that the underlying mechanisms appear to differ for the groups (i.e., impulsivity vs. passivity) but that the everyday life consequences of having a dual disorder are the same: both groups get caught in a vicious circle of symptoms and substance use. Our findings show SUD co-occurring with ADHD or ASD to be associated with EF impairments and thus have consequences for the daily life functioning and social interactions of patients. To help patients cope with these cognitive deficits, their treatment and care should be aimed at the areas of time management, organization, problem solving, self-control, motivation, and the regulation of emotions [27]. An integrated cognitive behaviour therapy protocol [28] possibly in combination with atomoxetine [29] or high doses of methylphenidate [30] appears promising for the treatment of SUD + ADHD. Further research is nevertheless needed in these areas. To our knowledge, the treatment of co-occurring SUD + ASD has not yet been studied. Considering the negative consequences for the everyday lives of the patients, the treatment possibilities for this dual diagnosis group should be studied.

Further examination of the actual experiences of patients is needed to provide a clear basis for their treatment. The treatment and care experiences of patients with ADHD or ASD and a co-occurring SUD should be examined, for example, in order to gain insight into their opinions about what contributes to their recovery and which coping strategies should be reinforced and stimulated.
